# Prevention of Fine Dust-Induced Vascular Senescence by *Humulus lupulus* Extract and Its Major Bioactive Compounds

**DOI:** 10.3390/antiox9121243

**Published:** 2020-12-07

**Authors:** Saugat Shiwakoti, Deepak Adhikari, Jeong Pyo Lee, Ki-Woon Kang, Ik-Soo Lee, Hyun Jung Kim, Min-Ho Oak

**Affiliations:** 1 College of Pharmacy, Mokpo National University, Jeonnam, Muan-gun 58554, Korea; saugat.shiwakoti04@gmail.com (S.S.); dpak7adh@gmail.com (D.A.); escape12@mokpo.ac.kr (J.P.L.); 2 Division of Cardiology, Eulji University Hospital, Eulji University School of Medicine, Daejeon 34824, Korea; kwkang@eulji.ac.kr; 3 College of Pharmacy, Chonnam National University, Gwangju 61186, Korea; islee@chonnam.ac.kr

**Keywords:** *Humulus lupulus*, xanthohumol, isoxanthohumol, endothelial senescence, fine dust, air pollution, oxidative stress, endothelial dysfunction

## Abstract

Both short- and long-term exposure to fine dust (FD) from air pollution has been linked to various cardiovascular diseases (CVDs). Endothelial cell (EC) senescence is an important risk factor for CVDs, and recent evidence suggests that FD-induced premature EC senescence increases oxidative stress levels. Hop plant (*Humulus lupulus*) is a very rich source of polyphenols known to have nutritional and therapeutic properties, including antioxidant behavior. The aims of this study were to evaluate whether *Humulus lupulus* extract prevents FD-induced vascular senescence and dysfunction and, if so, to characterize the underlying mechanisms and active components. Porcine coronary arteries and endothelial cells were treated with FD in the presence or absence of hop extract (HOP), and the senescence-associated-beta galactosidase (SA-β-gal) activity, cell-cycle progression, expression of senescence markers, oxidative stress level, and vascular function were evaluated. Results indicated that HOP inhibited FD-induced SA-β-gal activity, cell-cycle arrest, and oxidative stress, suggesting that HOP prevents premature induction of senescence by FD. HOP also ameliorated FD-induced vascular dysfunction. Additionally, xanthohumol (XN) and isoxanthohumol (IX) were found to produce the protective effects of HOP. Treatment with HOP and its primary active components XN and IX downregulated the expression of p22^phox^, p53, and angiotensin type 1 receptor, which all are known FD-induced redox-sensitive EC senescence inducers. Taken together, HOP and its active components protect against FD-induced endothelial senescence most likely via antioxidant activity and may be a potential therapeutic agent for preventing and/or treating air-pollution-associated CVDs.

## 1. Introduction

Air pollution consists of a heterogeneous mixture of gases, liquids, and particulate matter. Among these components, particulate matter has been shown to have the most adverse health effects [1]. Fine dust (FD) is a form of air pollution consisting of a mixture of solid and liquid particles suspended in air, which varies in size and chemical composition. Numerous epidemiological studies have associated FD with an increase in cardiovascular mortality and morbidity [2,3]. Both short-term and long-term exposure to air pollution increases the risk of cardiovascular disease (CVD), at least in part by promoting systemic oxidative stress and inflammation, as well as autonomic nervous system imbalance [2,4]. Recent studies proposed that air pollution induces endothelial dysfunction, which is the major risk factor for CVD [5,6].

Endothelial cell (EC) senescence contributes to endothelial dysfunction [7,8] and is an important risk factor for the development of CVD during aging [9]. Vascular EC senescence can be induced prematurely through a variety of factors including oxidative stress, angiotensin II, high glucose, radiation, and DNA damage [10,11,12,13,14]. Recent evidence suggests that FD-induced premature senescence of ECs most likely occurs via activation of the redox-sensitive local renin–angiotensin system (LAS). Moreover, treatment with the antioxidant *N*-acetylcysteine (NAC) delayed FD-induced EC senescence and alleviated endothelial dysfunction [15].

Numerous studies have indicated that polyphenol-rich food or food-derived products, such as grape-derived products, teas, cocoa, and black and red berries, help decrease the occurrence of CVD via endothelial protection [16,17]. The protective cardiovascular effects of polyphenol-rich foods has been attributed to their antioxidant activities and several additional properties, including anti-inflammatory effect, prevention of low-density lipoprotein oxidation, inhibition of platelet aggregation and adhesion, and repression of smooth muscle cell migration and proliferation [18,19,20,21]. Recent investigations have also indicated that polyphenols are able to improve endothelial function by preventing endothelial senescence induced by different types of stimuli, including those promoting oxidation [17].

Hop (*Humulus lupulus* L., Cannabaceae) has proven to be very rich sources of polyphenols, which comprise 3–6% of the dry weight of hop cones [22]. The history of hops as a medicinal plant dates back to 2000 years [23]. In addition to their essential role in brewing beer, hops possess intriguing beneficial properties for human health, with nutritional and therapeutic effects including antioxidant, antiplatelet, anticancer, antiproliferative, and anti-inflammatory behaviors [24,25,26,27]. Hops are also a very rich source of potent phytochemicals such as proteins, lipids, amino acids, resins, and many essential oils [22,28].

As the threat of airborne FD particles causing cardiovascular complications associated with endothelial dysfunction rapidly grows, alternative approaches regarding the identification of potential therapeutic agents in the treatment of air-pollution-induced endothelial dysfunction and senescence must be investigated. The purpose of the current study was to investigate whether *H. lupulus* (hop) prevents FD-induced premature senescence of ECs and, if so, to characterize the underlying mechanisms and major bioactive compounds. 

## 2. Materials and Methods

### 2.1. FD

FD (ERM-CZ100)—a road dust originating from the Wislostor ada road tunnel in Warsaw, Poland—was purchased from Sigma-Aldrich (St. Louis, MO, USA). It is the dust collected from a tunnel in Warsaw, Poland. It is certified for the mass fraction of selected polycyclic aromatic hydrocarbons and contains different particle sizes. A stock solution of FD was prepared in dimethyl sulfoxide (DMSO). A working concentration of FD was prepared by further diluting a stock solution of FD in medium. The concentration of FD was always maintained equivalently throughout the experiment.

### 2.2. Preparation of Extract and Isolation of Hop Prenylflavonoids

The hop extract (HOP) used in the study was prepared from a commercial hop extract that was adsorbed on diatomaceous earth (Xantho-Extract^®^, Hopsteiner, Germany) [29]. The product (5 kg) was eluted with methanol on glass column to desorb hop constituents, and the methanol-soluble layer was evaporated at the reduced pressure to yield dark-greenish powder (931.6 g). The extract HOP was stored at −20 °C until use. Furthermore, two major hop prenylated flavonoids, isoxanthohumol (IX) and xanthohumol (XN), were isolated from another commercial hop resin extract (Hopsteiner, Mainburg, Germany). The resin extract (8.48 g) was dissolved in methanol and partitioned with the same volume of *n*-hexane to remove hydrophobic compounds. Then, the hydrophilic portion solved in methanol was subjected to flash chromatography (Biotage Isolera, Uppsala, Sweden) using a C18 cartridge (120 g) with an acetonitrile–H_2_O (0.1% formic acid) gradient system at 30 mL/min. The subfractions containing IX and XN were further purified with semipreparative high-performance liquid chromatography (HPLC) (Waters 600 system, Milford, MA, USA), using SunFire^TM^ Prep C18 OBD column (5 μm, 19 × 150 mm) with acetonitrile–H_2_O (0.1% formic acid) mixtures at a flow rate of 14.0 mL/min to give IX (22.3 mg) and XN (24.8 mg). The structures of compounds were identified by comparison with spectral data reported previously [30] (Figure S1, Spectral data for hop prenylated flavonoids isoxanthohumol (IX) and xanthohumol (XN), Supplementary Materials).

### 2.3. Cell Culture

Pig heart was collected from a local slaughterhouse (Mokpo, Jeonnam, South Korea) right after sacrifice and moved to the lab within 20 min, maintained at 4 °C in Krebs bicarbonate solution (mM: 119 NaCl, 4.7 KCl, 1.18 KH_2_PO_4_, 1.18 MgSO_4_, 1.25 CaCl_2_, NaHCO_3_, and 11 d-glucose (pH 7.4)). Porcine coronary ECs were isolated from the left atria of porcine immediately upon arriving at the lab as previously described [31]. Briefly, the left anterior descending coronary artery of the porcine heart was dissected and cleaned of loose connective tissue in oxygenated (95% O_2_ and 5% CO_2_) Krebs bicarbonate solution. Endothelial cells were isolated by collagenase treatment (Type-I, 1 mg/mL, Worthington Biochemical, Lakewood, NJ, USA) for 15 min at 37 °C and cultured in T flasks containing Dulbecco’s modified Eagle’s medium (DMEM) supplemented with penicillin (100 U/mL), streptomycin (100 U/mL), fungizone (250 μg/mL), and 10% fetal bovine serum (FBS) and grown for 48–72 h (passage (P)0). The medium was changed every 48 h. Porcine coronary ECs prepared from P0–P1 cultures were detached using trypsin and further passaged at a ratio of 1:3. Premature atrial EC senescence was induced by incubating cells (P1) with FD (30 and 100 μg/mL) diluted in growth medium for indicated times; untreated cells served as the control group. To investigate the effect of HOP and its bioactive components (XN, IX), porcine coronary ECs were treated with HOP (30 and 100 μg/mL) and different doses of XN and IX (10, 30, 100 µM) in the presence of 30 µg/mL FD.

### 2.4. Detection of Senescence-Associated β-Galactosidase Activity (SA-β-gal)

Senescence-associated β-galactosidase activity was observed on porcine coronary artery (PCA) and porcine coronary ECs by staining with X gal solution (citric acid/NaH_2_PO_4_ (1×), potassium ferricyanide (5 mM), potassium ferrocyanide (5 mM), NaCl (150 mM), MgCl_2_ (2 mM), X gal (1 mg/mL)) [32]. Briefly, porcine coronary ECs on P1 were treated with FD (30 µg/mL) alone or with hop and different concentrations of XN and IX for 24 h and then stained with X gal solution overnight. After staining, the cells were washed with phosphate-buffered saline (PBS) and methanol before viewing under a bright-field microscope. The proportion of cells positive for SA-β-gal activity were easily determined by counting the number of blue cells in the total population.

For tissue, PCA rings, after incubation for 24 h with FD (100 µg/mL) alone or with hop and different concentrations of XN and IX, were fixed using 4% formaldehyde and then stained with X gal solution overnight. The frozen section of the rings was made in optimum cutting temperature solution, and thin sections (10 μM) were sliced using a microtome. The tissue was placed on a glass slide and mounted with FluorSave medium. Finally, the tissue was covered with a coverslip and viewed under a bright-field microscope.

### 2.5. Proliferation Assay

Porcine coronary ECs were seeded in a 96-well plate at a density of 1 × 10^4^ cells/well and cultured for 12 h in 10% FBS/DMEM, then starved overnight in 0.1% FBS. For the proliferation assay, cells were treated with FD alone or in combination with different concentrations of HOP, XN, and IX. The control group was treated with medium without FD. After adding 3-(4,5-dimethylthiazol-2-yl)-2,5-diphenyl tetrazolium bromide (MTT) (0.1 mg/mL) for 4 h, formazan crystals that formed were solubilized in 100 μL dimethylsulfoxide (DMSO), and the absorbance was measured at 540 nm.

### 2.6. Western Blot Analysis

Cells were homogenized and lysed in radioimmunoprecipitation assay (RIPA) buffer (Cell Signaling Technology, Danvers, MA, USA), supplemented with protease and phosphatase inhibitor cocktail (Sigma-Aldrich, St. Louis, MO, USA). The cell lysate was centrifuged at 13,000 rpm at 4 °C for 20 min. The supernatant was collected and quantified using albumin standard (GenDEPOT, Katy, TX, USA; cat. no. A1100-055) and DC protein assay reagent (Bio-Rad, Hercules, CA, USA). A total of 15 µg per well of protein was boiled with 1× loading buffer (Thermo Fisher Scientific, Waltham, MA, USA; cat. no. 39000) for 10 min. The protein was loaded onto a 10% sodium dodecyl sulfate–polyacrylamide gel and separated by electrophoresis, then transferred to a polyvinylidene difluoride membrane that was blocked with 5% bovine serum albumin (BSA) for 1 h and incubated overnight at 4 °C with primary antibodies against β-actin (cat. no. 2125, 1:5000) (Cell Signaling Technology), p53 (cat. no. SC-6243, 1:1000), p22phox (sc-271968, 1:1000), and AT_1_ (cat. no. SC-515884, 1:1000) (From Santa Cruz Biotechnology, Dallas, TX, USA), diluted in 5% BSA. After washing, the membrane was incubated with horseradish peroxidase (HRP)-conjugated donkey anti-rabbit immunoglobulin G (IgG) (Cell Signaling Technology, cat. no. 7074S) or HRP-conjugated anti-mouse IgG diluted 1:5000 (Cell Signaling Technology, cat. no. 7076S), then washed and treated with enhanced chemiluminescence substrate (GE Healthcare, Little Chalfont, UK; cat. no. RPN2232) and visualized with a chemiluminescence system (UVItec, Cambridge, UK).

### 2.7. Measurement of Intracellular Reactive Oxygen Species (ROS) Levels in Porcine Coronary ECs

A fluorometric microplate assay was used for the detection of oxidative stress by detecting oxidation of 2′,7′-dichlorofluorescein-diacetate (DCF-DA) into the highly fluorescent compound 2′,7′-dichlorofluorescein (DCF) due to the presence of ROS [33]. Porcine coronary ECs were seeded at a concentration of 1 × 10^4^ cells/well in black 96-well clear flat-bottomed plates and allowed to adhere overnight. Cells were then treated with DCF-DA dissolved in DMSO (20 mM final concentration) and incubated for 30 min at 37 °C, 5% CO_2_, and 90% humidity. After washing with PBS, cells were treated with FD alone or in combination with various concentrations of HOP, XN, and IX for 24 h. The fluorescence intensity of the oxidation product was measured at an excitation/emission wavelength of 485/535 nm.

Intracellular ROS levels were also evaluated using redox-sensitive fluorescent dye, dihydroethidium (DHE). Cells were seeded at a concentration of 5 × 10^4^ cells/well in six-well plates and allowed to adhere overnight. Cells were then treated with FD alone or in combination with various concentrations of HOP and XN/IX for 24 h. DHE dissolved in DMSO was added to cells at 10 μM. After incubation for 30 min at 37 °C under 5% CO_2_ atmosphere, the cells washed with PBS were visualized under a fluorescence microscope.

### 2.8. Vascular Reactivity Study

Pig hearts were collected from a local slaughterhouse, and vascular reactivity was assessed as indicated previously [31]. Briefly, the dissected left anterior descending coronary artery of the porcine heart was cleaned of loose connective tissue in oxygenated (95% O_2_ and 5% CO_2_) Krebs bicarbonate solution, and then cut into rings (3–4 mm in length) that were incubated in 10% FBS/DMEM in an atmosphere of 95% O_2_ and 5% CO_2_ for 24 h. To investigate the protective effects of HOP and XN/IX, porcine aortic rings were pretreated in FD along with HOP (100 ug/mL) and XN/IX (100 µM), respectively for 24 h. Rings of porcine coronary arteries were then suspended in organ baths containing oxygenated (95% O_2_ and 5% CO_2_) Krebs bicarbonate solution at 37 °C to assess changes in isometric tension. After equilibrating for 90 min under a resting tension of 5 g, the rings were contracted twice by applying 80 mM KCl. Thereafter, rings were contracted with U46619 (a thromboxane A2 receptor agonist) to 80% of maximum contraction, and relaxation in response to bradykinin (0.3 μM) was determined.

### 2.9. Statistical Analysis

Results are presented as the mean ± standard error of the mean (SEM) and were analyzed by one-way analysis of variance (ANOVA) with a Tukey post hoc test. Results obtained in the vascular reactivity study were analyzed by two-way ANOVA with a Bonferroni post hoc test. All statistical analyses were performed using Prism software (GraphPad Inc., La Jolla, CA, USA). A *p*-value < 0.05 was considered significant.

## 3. Result and Discussion

### 3.1. Hop Extract (HOP) Prevents FD-Induced Premature Endothelial Senescence and Dysfunction in Porcine Coronary Artery

**Figure 1:**
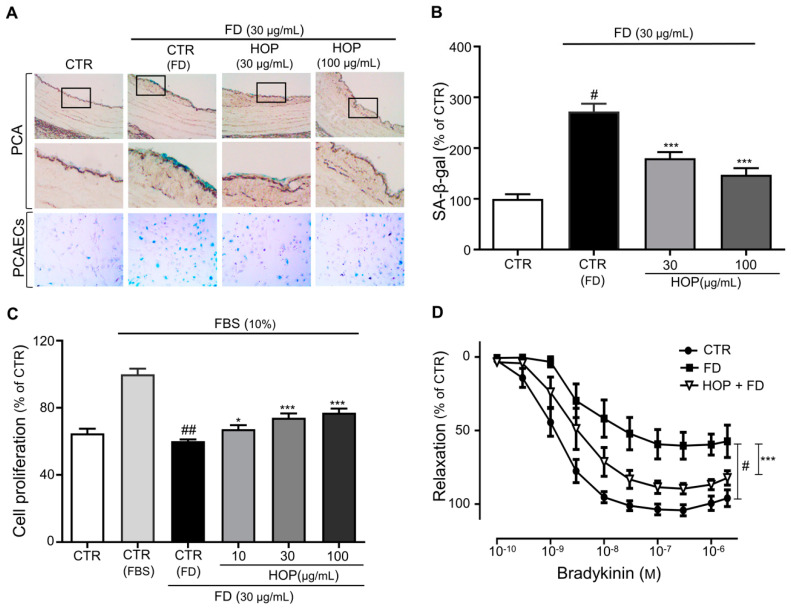
Hop extract (HOP) prevents fine dust (FD)-induced premature senescence. (**A**) Porcine coronary artery (PCA) rings and PCA endothelial cells (PCAECs) were incubated in the presence of FD or FD with either 30 or 100 μg/mL HOP before determination of senescence-associated β-galactosidase (SA-β-gal) activity by X gal staining. Representative images of SA-β-gal staining of PCA rings and PCAECs. (**B**) Cumulative data of SA-β-gal activity as a percentage of control. Data are expressed as the mean ± standard error of the mean (SEM) (*n* = 5); ^#^ *p* < 0.05 vs. control (CTR); *** *p* < 0.001 vs. FD alone (CTR-FD). (**C**) Dose-dependent increases in cell proliferation upon FD with HOP (10, 30, or 100 μg/mL) treatment. Data are expressed as the mean ± SEM (*n* = 6); ^##^ *p* < 0.01 vs. fetal bovine serum (FBS) alone (CTR-FBS); * *p* < 0.05, *** *p* < 0.001 vs. FD alone (CTR-FD). (**D**) Concentration–relaxation curves of FD-exposed aortic rings treated with 100 μg/mL HOP in response to bradykinin. Data are expressed as the mean ± SEM (*n* = 7–10); ^#^ *p* < 0.05 vs. control (CTR); *** *p* < 0.001 vs. FD alone (FD).

Previous studies suggested that FD induces premature EC senescence associated with endothelial dysfunction [15,34,35]. In this study, we investigated whether hop extract (HOP) affects FD-induced premature EC senescence. EC senescence was assessed on the basis of senescence-associated (SA)-β-gal activity indicated by levels of X gal staining in porcine coronary arteries (PCAs) and PCA endothelial cells (PCAECs). PCAs and PCAECs were incubated with FD alone or in combination with various concentrations of HOP. Consistent with a previous report, FD increased SA-β-gal activity in both PCAs and PCAECs (Figure 1A,B), which indicates that FD induces premature endothelium senescence. Treatment with HOP repressed the increase in SA-β-gal activity by FD in a concentration-dependent manner (Figure 1A,B), suggesting that HOP prevents FD-induced premature endothelial senescence.

The characteristic features of senescent cells are their limited number of cell divisions and reduced proliferation [36,37]. It has also been reported that FD induces cell-cycle arrest and reduces proliferation [38]. Thus, we studied whether HOP prevents an FD-induced reduction in ECs proliferation. Consistent with a previous study, the proliferation capacity of FD-exposed ECs was reduced compared to untreated ECs (Figure 1C). However, HOP application ameliorated FD-induced decreases in ECs proliferation in a dose-dependent manner.

The development and progression of EC senescence involve endothelial dysfunction that can result in various cardiovascular complications [39]. Similarly, exposure to FD is often followed by a rapid increase in ROS generation and, thus, impaired endothelial-dependent vasodilation and reduced bioavailability of endothelial nitric oxide (NO), contributing to endothelial dysfunction [40]. To test whether vascular dysfunction induced by FD can be prevented by HOP, coronary artery rings were incubated in 10% FBS/DMEM with or without HOP in the presence of FD and the vascular reactivity was evaluated. FD reduced vasorelaxation in response to bradykinin while treatment with HOP significantly increased the maximal relaxation, indicating that FD-induced endothelial dysfunction was prevented by components in HOP (Figure 1D).

Taken together, our results suggest that HOP prevents FD-induced EC senescence and ameliorates FD-induced decreases in cell proliferation. Senescent cells become enlarged and flattened and exhibit increases in senescence-associated β-galactosidase (SA-β-gal) activity [32]. SA-β-gal activity reflects an increase in lysosomal mass during EC aging and serves as a marker for senescence [41]. Our study showed that FD treatment led to EC senescence, as evidenced by enhanced SA-β-gal activity, with this effect reduced in PCAs and PCAECs treated with HOP. The functional changes associated with cellular senescence are also involved in various age-related vascular disorders, including endothelial dysfunction [39,42]. It was previously reported that exposure to air pollutants impairs endothelium-dependent vasodilation and decreases the bioavailability of endothelial NO, resulting in endothelial dysfunction [40]. Our results showed that treatment with HOP significantly increases the maximal relaxation to the FD-treated aortic rings, suggesting that HOP may prevent endothelial dysfunction associated with FD.

### 3.2. Xanthohumol and Isoxanthohumol in HOP Prevent FD-Induced Endothelial Premature Senescence

**Figure 2:**
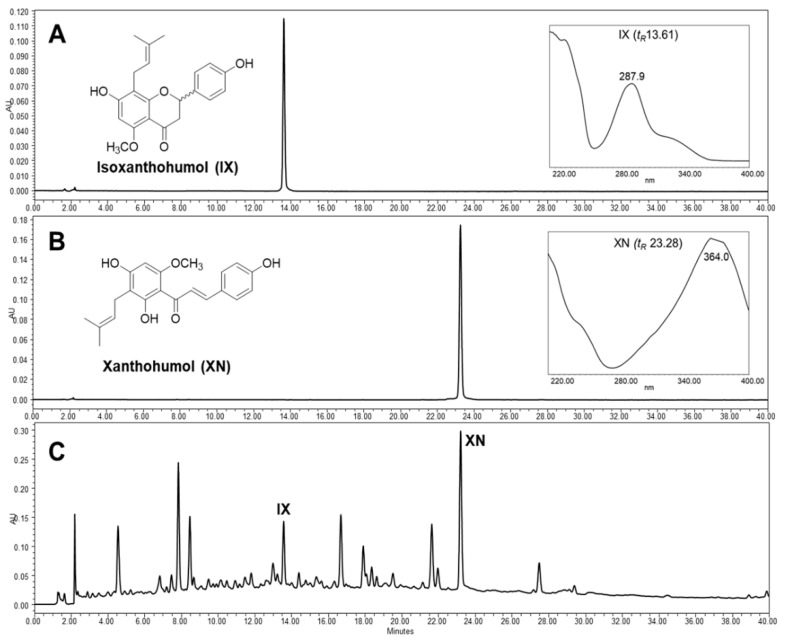
HPLC profiles for prenylated flavonoids and extract from hops: (**A**) isoxanthohumol (IX, 100 μg/mL), (**B**) xanthohumol (XN, 100 μg/mL), and (**C**) hop methanol extract (20 mg/mL). HPLC experiments were carried out using a Waters 1525 binary pump, 2707 autosampler, and 2998 photodiode array detector (PDA) detector with a SunFire C18 column (5 μm, 4.6 × 150 mm, Waters). The chromatographic conditions were as follows: acetonitrile (**A**) and 0.1% formic acid–water (**B**), with 25% to 100% of solvent A for 40 min at flow rate of 1.0 mL/min under 315 nm. Ultraviolet (UV) spectra were reported within a wavelength range of 210–400 nm.

The chemical profile of HOP was investigated using a high-performance liquid chromatogram with a photodiode array detector (HPLC–PDA). Two major prenylated flavonoids, isoxanthohumol (IX) and xanthohumol (XN), derived from HOP were detected at 315 nm (Figure 2). These compounds were isolated by preparative HPLC followed by spectroscopic analyses to elucidate their structures, with compounds identified by comparing these results with spectral data reported in the literature [30]. XN is the most abundant prenylated flavonoid with a chalcone scaffold in hop cones (0.1–1% of dry weight) and IX is the most abundant isomeric flavonoid of XN, which occurs naturally over time or produced by the brewing process [43]. XN and IX are considered to be the most important hop prenylflavonoids of hops and beer. There has been considerable recent biological and pharmacological interest in the possible applications of these compounds, including as cancer chemopreventatives, antioxidants and estrogenic treatments [43,44].

**Figure 3:**
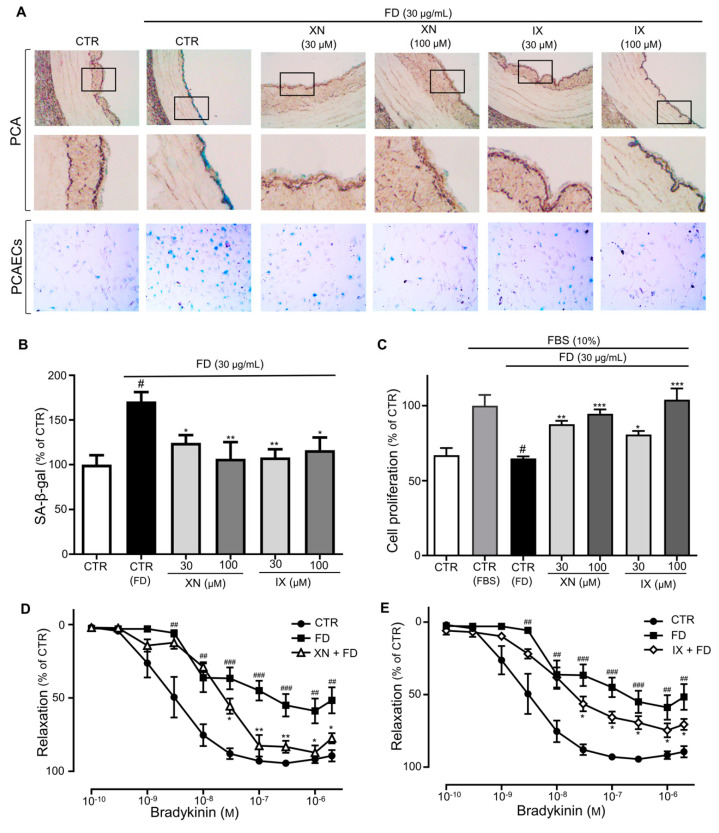
XN and IX prevent FD-induced premature senescence. (**A**) PCA rings and PCAECs were incubated in FD alone or FD in combination with XN or IX (30, 100 μg/mL) before determination of SA-β-gal activity by X gal staining. Representative images of SA-β-gal-stained PCA rings and PCAECs. (**B)** Cumulative data of SA-β-gal activity as a percentage of the control. Data are expressed as the mean ± SEM (*n* = 6); ^#^ *p* < 0.05 vs. CTR; * *p* < 0.05, ** *p* < 0.01 vs. FD alone (CTR-FD). (**C**) Dose-dependent increases in cell proliferation after FD and XN/IX (10,30 μM) treatment. Data are expressed as the mean ± SEM (*n* = 6); ^#^ *p* < 0.05 vs. FBS alone (CTR-FBS); * *p* < 0.05, ** *p* < 0.01, *** *p* < 0.001 vs. FD alone (CTR-FD). (**D**,**E**) Concentration–relaxation curves of FD-exposed aortic rings treated with XN and IX (100 μM) in response to bradykinin. Data are expressed as the mean ± SEM (*n* = 7–10); ^##^ *p* < 0.01, ^###^ *p* < 0.001 vs. CTR; * *p* < 0.05, ** *p* < 0.01 vs. FD alone (FD).

The protective effect of these major compounds, XN and IX, on FD-induced endothelial senescence was investigated. Treatment with XN and IX inhibited increases in SA-β-gal activity triggered by FD in PCAECs and PCAs (Figure 3A and 3B) and ameliorated FD-induced decreases in EC proliferation (Figure 3C). The vascular reactivity study indicated that bradykinin caused dose-dependent vasorelaxation, which was significantly reduced in the FD incubation compared with the control (median effective dose (ED_50_) of bradykinin was 3.3 ± 3.4 nM and 382.5 ± 9.6, while E_max_ was 95.7% ± 5.5% and 61.9% ± 8.4%, in the control and FD incubation, respectively; Figure 3D,E). The XN and IX treatment prevented the inhibitory effect of FD on bradykinin-induced relaxation (ED_50_ of bradykinin was 26.8 ± 4.7 nM and 42.2 ± 5.8 nM, while E_max_ was 91.9% ± 5.1% and 74.9% ± 6.1% in the FD plus XN and IX, respectively; Figure 3D,E). Taken together, our results showed that endothelial senescence induced by FD was significantly repressed by both XN and IX, indicating their protective role in alleviating endothelial dysfunction. These findings suggest that the endothelial protective effects of HOP may be, at least in part, due to presence of these active compounds.

### 3.3. HOP and Its Major Components XN and IX Decrease FD-Induced Oxidative Stress Levels

**Figure 4:**
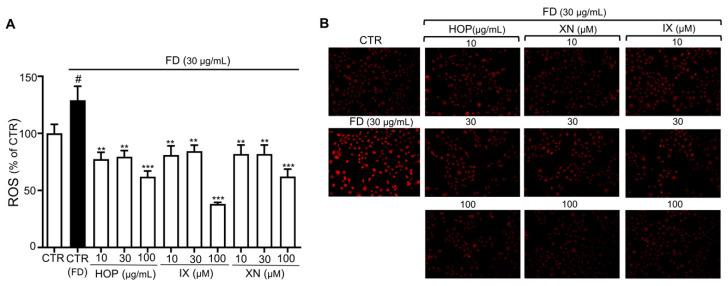
HOP and its major compounds XN/IX prevent FD-induced premature senescence by reducing oxidative stress in ECs. (**A**) Reactive oxygen species (ROS) levels were determined by measuring relative 2′,7′-dichlorofluorescein-diacetate (DCF-DA) fluorescence intensity and were calculated as a percentage of the control (CTR). Data are expressed as the mean ± SEM (*n* = 5); ^#^ *p* < 0.05 vs. control (CTR); ** *p* < 0.01, *** *p* < 0.001 vs. FD alone (CTR-FD). (**B**) Representative figure of fluorescence microscopy of redox-sensitive fluorescent dye (dihydroethidium, DHE) staining of live adherent passage 1 (P1) ECs in presence of FD alone or in combination with HOP (10, 30 μg/mL) and XN/IX (10, 30 μM respectively).

An imbalance between ROS generation and cellular antioxidant function is the main cause of aging and vascular damage resulting from endothelial dysfunction [45,46]. In addition, FD has been shown to increase oxidative stress, which causes endothelial senescence and dysfunction, with antioxidants such as NAC preventing FD-induced activation of redox-sensitive LAS and endothelial senescence [47]. To determine whether the antioxidant properties of HOP and its active components influence FD-induced endothelial senescence and dysfunction, we measured ROS levels using two different redox-sensitive fluorescent dyes, 2′,7′-dichlorofluorescein-diacetate (DCF-DA) and dihydroethidium (DHE). When DCF-DA diffuses into a cell, it is oxidized by ROS into DCF, which is highly fluorescent and, thus, enables measurement of general ROS activity within the cell [33]. Exposure to FD for 24 h increased DCF fluorescence, indicating elevated oxidative stress, which is consistent with previous work (Figure 4A) [48]. Treatment with HOP and its active compounds (XN, IX) decreased FD-induced oxidative stress (Figure 4A). Moreover, oxidative stress was strongly reduced compared with control by HOP and IX/XN at concentrations of 100 g/mL and 100 M, respectively. Results obtained using another ROS indicator, DHE, which freely permeates cell membranes and mainly reports superoxide and hydrogen peroxide levels, was consistent with those obtained using DCF-DA (Figure 4B). Taken together, HOP and its active components (XN, IX) prevent FD-induced premature senescence, at least in part, via their antioxidant properties [49].

Oxidative stress occurs when ROS production is not properly balanced by cellular antioxidant functions, which is the main cause of vascular dysfunction [46]. Particles such as transition metals and polyaromatic hydrocarbon quinones present in FD are potent oxidants and are themselves capable of ROS generation upon interaction with biological specimens [47]. In addition, FD has been previously reported to induce premature EC senescence and endothelial dysfunction at least in part by increasing oxidative stress in redox-sensitive local angiotensin systems [15]. A reduction in oxidative stress could, thus, be a major strategy for preventing senescence, and antioxidant therapy could positively affect and delay the onset of air-pollution-induced cardiovascular diseases [50].

Various antioxidants, such as ascorbic acid and *N*-acetylcysteine, have shown a significant role in delaying age-related endothelial dysfunction [51,52]. Similarly, numerous epidemiological studies have shown that natural antioxidants, such as polyphenols from tea, cacao, berries, and other plants, can trigger increases in the formation of vasoprotective factors such as nitric oxide (NO) in ECs and, thus, prevent endothelial dysfunction. Polyphenol induces transcription-mediated signaling, thereby activating endogenous antioxidant enzymatic defense mechanisms that exert longer-lasting effects compared to other antioxidants [53]. Thus, the role of polyphenolic compound to protect endothelial dysfunction has been proven to be beneficial [17,54].

Hops are a major source of phenolic compounds, and numerous animal and clinical experiments have asserted that the antioxidant properties of hops are related to the beneficial effects of their polyphenols [22]. Among the hop prenylflavonoids, XN and IX have been shown to have significant antioxidant potential [55,56,57,58]. The direct radical scavenging ability of XN and IX varies depending on the experimental methods. Tronina et al. reported a 1,1-diphenyl-2-picrylhydrazyl (DPPH) radical-scavenging activity of XN and IX with a half maximal inhibitory concentration (IC_50_) value of 1.98 ± 0.24 µM and 35.42 ± 0.11 µM, respectively (0.52 ± 0.2 µM, IC_50_ of ascorbic acid) [59]. However, XN was about ninefold and threefold more potent than Trolox in scavenging physiologically relevant ROS such as hydroxy and peroxyl radicals in ORAC assay [60]. IX was equally effective to Trolox, but more potent than XN at a high concentration [60]. Although most investigations suggest that IX is a weaker direct radical scavenger than XN, a pretreatment with IX clearly reduced oxidative stress in vivo [61], suggesting that the activity of XN and IX is not only due to a direct radical-scavenging effect, but due to a modulation of the antioxidant defense system of cells. In this study, HOP and its major components, XN and IX, exhibited potent antioxidant effects; therefore, their antioxidant properties could play prime roles in preventing FD-induced senescence by alleviating oxidative stress. We, thus, conducted further studies to examine the mechanisms underlying the protective effects of HOP, XN, and IX.

### 3.4. HOP and Its Major Compounds XN and IX Downregulate Redox-Sensitive Endothelial Senescence Markers

We further assessed the effects of HOP, IX, and XN on senescence-marker-associated molecular pathways to confirm whether their antioxidant properties prevent FD-induced vascular aging and dysfunction.

**Figure 5:**
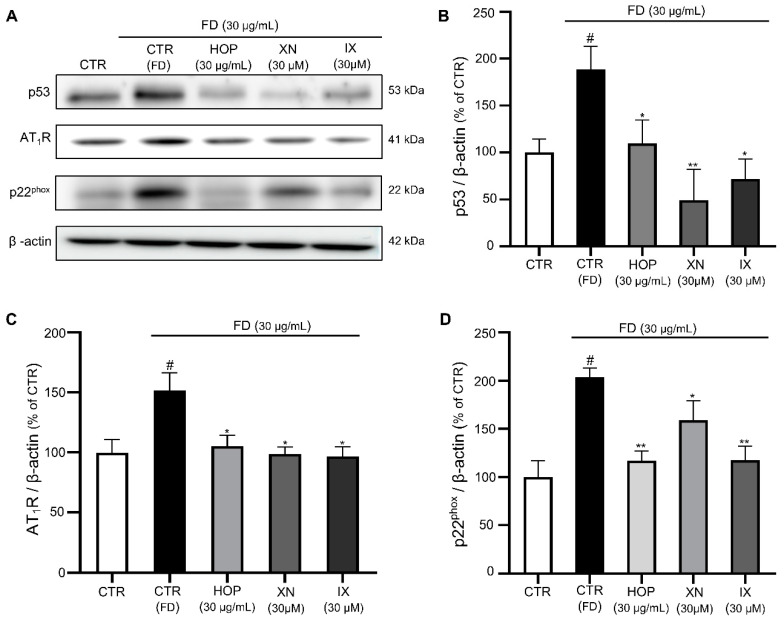
HOP and its active compounds XN and IX reduce the expression of p53, AT_1_R, and p22^phox^. (**A**) Representative blots of p53, AT_1_R, and p22^phox^ proteins of ECs in presence of FD alone or in combination with HOP extract and XN/IX. (**B–D**) Corresponding cumulative data of p53, AT_1_R, and p22^phox^, respectively. Data are expressed as the mean ± SEM (*n* = 3); ^#^ *p* < 0.05 vs. control (CTR); * *p* < 0.05, ** *p* < 0.01 vs. FD alone (CTR-FD).

Endothelial senescence is characterized by irreversible growth arrests and alterations of gene expression level. Various stimuli such as DNA damage, high glucose, radiation, telomere shortening, and oncogenic stress trigger senescence programs in ECs [10,11,12,13,14]. The transcription factor p53 plays a critical role in cellular responses to these stresses, and its activation leads to cell growth arrest, allowing for DNA repair, or triggers cellular senescence [62]. A previous study reported that FD-induced senescence in ECs is associated with increased expression of p53 [15]. Similarly, in our work, treatment with FD increased the expression level of p53, while treatment with HOP, XN, and IX significantly reduced the expression of p53, supporting a protective role for HOP, XN, and IX toward FD-induced senescence (Figure 5A,B).

Multiple lines of evidence indicate that ROS-generating enzymes are the causal link between the excess formation of ROS and EC senescence [63]. It was previously reported that age-related increases in ROS formation involve nicotinamide adenine dinucleotide phosphate (NADPH) oxidase, a major cellular source of ROS in cardiovascular disease [64]. In addition, endothelial senescence is associated with upregulation of the expression of p22^phox^, a component of NADPH oxidase, which increases superoxide levels. Moreover, chronic exposure to FD induces vascular dysfunction through NADPH activation in animal models [12,65]. Therefore, the expression level of the NADPH oxidase subunit p22^phox^ in the presence of HOP, XN, and IX was assessed. FD increased p22^phox^ expression, while HOP, XN, and IX reduced p22^phox^ expression, indicating that repression of FD-induced oxidative stress and vascular senescence by HOP and its components are, as least in part, associated with the downregulation of NADPH oxidase (Figure 5A,D). In the endothelium, ROS are generated by various enzymes such as NADPH oxidase, xanthine oxidase, and uncoupled endothelial nitric oxide synthase (eNOS), with NADPH oxidase enzymes considered to be the primary source of ROS in cardiovascular tissue [66]. Excessive ROS production by these enzymes results in increased NO degradation, which helps trigger the onset of endothelial dysfunction [46]. A recent study showed that pharmacological blocking of these ROS-producing enzymes with certain inhibitors reduces oxidative stress and delays senescence in ECs [67]. Another study done with polyphenols found that the antioxidant effects of polyphenols are characterized by their abilities to suppress the activity of specific ROS-generating enzymes such as NADPH oxidase [68]. Since hops are a rich source of polyphenols, their antioxidant activity may suppress NADPH oxidase activity, thus reducing free-radical formation.

Studies have also suggested the involvement of the LAS as a potent inducer of vascular oxidative stress in senescent ECs [69]. Previous work demonstrated the role of LAS signaling in senescence promotion, contributing to endothelial dysfunction [70]. Thus, we investigated whether HOP, IX, and XN inhibit the overexpression of LAS components in aging cells. AT_1_R was upregulated in FD-exposed ECs compared to untreated ECs. Treatment with HOP and IX/XN prevented upregulation of AT_1_R in FD-exposed ECs, suggesting that HOP, IX, and XN prevent senescence-associated activation of redox-sensitive LAS components (Figure 5A,C). Animal and cell culture experiments have shown that angiotensin II increases ROS formation by NADPH oxidases. Angiotensin II directly stimulates NADPH oxidase activity, which further results in the redox-dependent activation of ROS production by other cellular sources [71]. EC senescence has been shown to stimulate AT_1_ receptor activity [72], and treatment with AT_1_ receptor blockers has protective effects against endothelial senescence [73]. Our result also showed that HOP and its active compounds (XN/IX) prevent upregulation of AT_1_R, thus preventing endothelial senescence associated with redox-sensitive LAS activation.

## 4. Conclusions

Our results demonstrated that hop extract and its active compounds (XN and IX) prevent FD-induced premature EC senescence, at least in part, by reducing the oxidative stress mediated by NADPH oxidase and LAS, as shown by downregulation of p22 ^phox^ and AT_1_R expression, respectively. Thus, the antioxidant properties of hop extract could play a major role in preventing FD-induced senescence and vascular dysfunction. We, thus, accomplished the proposed objectives, including extraction and isolation of its active compounds XN and IX, which highlights the need for more research in order to understand the bioavailability of specific molecules and their potential mechanisms for the future use of hops as a potential therapeutic agent in the treatment of vascular complications associated with EC senescence.

## Supplementary Materials

The following are available online at https://www.mdpi.com/2076-3921/9/12/1243/s1, Figure S1: Spectral data for hop prenylated flavonoids isoxanthohumol (IX) and xanthohumol (XN).
