# Supplementary material for: Prevention of Fine Dust-Induced Vascular Senescence by Humulus lupulus Extract and Its Major Bioactive Compounds
Source: Antioxidants (Basel). 2020 Dec 7;9(12):1243. doi: 10.3390/antiox9121243 (PMC7762380; doi:10.3390/antiox9121243)
Supplement: Supplementary file 1 [file antioxidants-09-01243-s001.pdf]

Supplementary Information

Prevention of Fine Dust–Induced Vascular Senescence by *Humulus lupulus* Extract and Its Major Bioactive Compounds

Saugat Shiwakoti [1], Deepak Adhikari [1], Jeong Pyo Lee [1], Ki-Woon Kang [2], Ik-Soo Lee [3], Hyun Jung Kim [1,*] and Min-Ho Oak[1,*]

[1] College of Pharmacy, Mokpo National University, Jeonnam 58554, Korea
[2] Division of Cardiology, Eulji University Hospital, Eulji University School of Medicine, Daejeon, 34824, Republic of Korea
[3] College of Pharmacy, Chonnam National University, Gwangju 61186, Korea
* Correspondence: mhoak@mokpo.ac.kr; Tel.: +82-61-450-2681 (M.-H.O.) and hyunkim@mokpo.ac.kr; Tel.: +82-61-450-2686 (H.J.K.)

Figure S1. Spectral data for hop prenylated flavonoids isoxanthohumol (IX) and xanthohumol (XN).

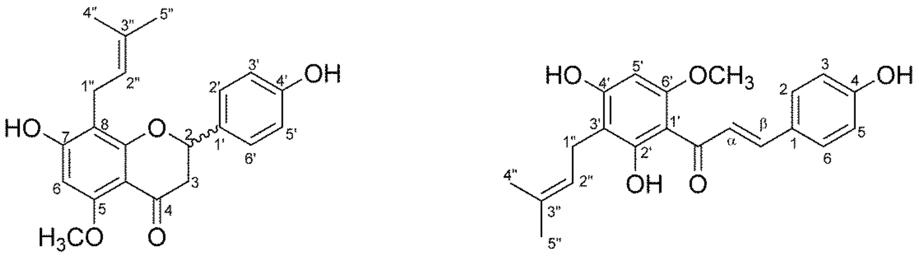

isoxanthohumol (IX) xanthohumol (XN)

Figure S1. Spectral data for hop prenylated flavonoids isoxanthohumol (IX) and xanthohumol (XN).

The ^{1}H and ^{13}C nuclear magnetic resonance (NMR) data of IX and XN were carried out on a JEOL ECZ-500R F (ourier transform-nuclear magnetic resonance spectrometer (JEOL, Tokyo, Japan). Liquid chromatography–mass spectrometry (LC–MS) data were obtained by a by Agilent 6120 quadruple mass spectrometry (MS) system coupled to Agilent 1260 Infinity liquid chromatography (LC) (Agilent Technologies, Santa Clara, CA, USA) at positive and negative electrospray ionization (ESI) mode.

Isoxanthohumol (IX), ^{1}H NMR (CD_3OD, 500 MHz) : δ 7.28 (2H, d, *J* = 8.5 Hz, H-2′,6′), 6.79 (2H, d, *J* = 8.5 Hz, H-3′,5′), 6.09 (1H, s, H-6), 5.25 (1H, dd, *J* = 12.5, 3.0 Hz, H-2), 5.11 (1H, brt, *J* = 6.8 Hz, H-2′′), 3.77 (3H, s, 5-OCH_3), 3.18 (2H, m, H-1′′), 2.95 (1H, dd, *J* = 16.5, 13.0 Hz, H-3a), 2.63 (1H, dd, *J* = 16.5, 3.0 Hz, H-3b), 1.59 (3H, s, H-5′′), 1.53 (3H, s, H-4′′). ^{13}C NMR (CD_3OD, 125 MHz) δ 191.6 (C–4), 162.9 (C-8a) 162.5 (C-7), 160.5 (C-5), 157.5 (C-4′), 130.3 (C-3′′), 130.2 (C-1′), 127.5 (C-2′,6′), 122.6 (C-2′′), 114.9 (C-3′,5′), 108.6 (C-8), 104.5 (C-4a), 92.1 (C-6), 78.7 (C-2), 54.6 (5-OCH_3), 44.9 (C-3), 24.6 (C-5′′), 21.4 (C-1′′), 16.6 (C-4′′). ESIMS *m*/*z* 355.1 $[M + H]^+$ (calcd. for $C_{21}H_{23}O_5$).

Xanthohumol (XN), ^{1}H NMR (CD_3OD, 500 MHz) : δ 7.77 (1H, d, *J* = 15.5 Hz, H-α), 7.64 (1H, d, *J* = 15.5 Hz, H- β), 7.47 (2H, d, *J* = 8.5 Hz, H-2,6), 6.80 (2H, d, *J* = 8.5 Hz, H-3,5) 6.00 (1H, s, H-5′), 5.17 (1H, brt, *J* = 7.0 Hz, H-2′′), 3.88 (3H, s, 6′-OCH_3), 3.20 (2H, *J*= 7.0 Hz, H-1′′), 1.74 (3H, s, H-5′′), 1.63 (3H, s, H-4′′). ^{13}C NMR (CD_3OD, 125 MHz) δ 192.7 (C–4), 164.8 (C-2′) 162.4 (C-4′), 161.1 (C-6′), 159.7 (C-4), 142.0 (C-β), 130.2 (C-3′′), 129.9 (C-2,6), 127.1 (C-1), 124.5 (C-α), 122.9 (C-2′′), 115.5 (C-3,5), 108.0 (C-3′), 105.2 (C-1′), 90.3 (C-5′), 54.6 (6′-OCH_3), 24.6 (C-5′′), 20.9 (C-1′′), 16.5 (C-4′′). ESIMS *m*/*z* 355.2 $[M + H]^+$ (calcd. for $C_{21}H_{23}O_5$).
